# Intelligent Analysis of Premature Ventricular Contraction Based on Features and Random Forest

**DOI:** 10.1155/2019/5787582

**Published:** 2019-10-07

**Authors:** Tiantian Xie, Runchuan Li, Shengya Shen, Xingjin Zhang, Bing Zhou, Zongmin Wang

**Affiliations:** ^1^School of Information Engineering, Zhengzhou University, Zhengzhou 450000, China; ^2^Cooperative Innovation Center of Internet Healthcare, Zhengzhou University, Zhengzhou 450000, China; ^3^College of Information & Business, Zhongyuan University of Technology, Zhengzhou 450000, China; ^4^State Key Laboratory of Mathematical Engineering and Advanced Computing, Zhengzhou 450000, China

## Abstract

Premature ventricular contraction (PVC) is one of the most common arrhythmias in the clinic. Due to its variability and susceptibility, patients may be at risk at any time. The rapid and accurate classification of PVC is of great significance for the treatment of diseases. Aiming at this problem, this paper proposes a method based on the combination of features and random forest to identify PVC. The RR intervals (pre_RR and post_RR), R amplitude, and QRS area are chosen as the features because they are able to identify PVC better. The experiment was validated on the MIT-BIH arrhythmia database and achieved good results. Compared with other methods, the accuracy of this method has been significantly improved.

## 1. Introduction

Electrocardiogram (ECG) is a graph that records the changes in electrical activity produced by each heart cycle of the heart from the body surface. It contains abundant basic functions and pathological information of the heart. Therefore, it is of great significance for the evaluation of cardiac safety and the evaluation of various treatment methods. It is also an important means for the examination and diagnosis of various arrhythmias. Premature ventricular contraction (PVC) is the most widespread and common arrhythmia in the clinic, and it represents the abnormal behaviour of signals produced by ECG. PVC is not immediately life-threatening, but it can cause a deadly heart rhythm [1, 2]. At present, for the diagnosis of the disease, doctors can only use the current medical technology for diagnosis based on their personal experience. At the same time, doctors may make a wrong diagnosis due to long hours of high-intensity work. However, the use of computer-aided detection of PVC can effectively improve the efficiency of diagnosis.

At present, machine learning has been widely used in medical diagnosis to help doctors improve the efficiency of diagnosis and treatment, so that doctors can diagnose diseases as soon as possible. For example, Liu et al. [3] proposed a method for diagnosing PVC based on the Lyapunov exponent. This method can distinguish PVC from other types by analyzing the Lyapunov exponent and training learning vector quantization. Gutiérrez-Gnecchi et al. [4] identified each waveform based on the quadratic wavelet transform process and identified eight heartbeat types through a probabilistic neural network. Although the method proposed in [3, 4] can identify PVC well, these high classification results which are based on small datasets or duplicate data are not tested on more datasets. Zarei et al.' study [5] was based on the “replacement” strategy to examine the effect of each heartbeat on the main direction change and proposed a method for detecting the newly arrived PVC heartbeat. Li et al. [6] proposed a method for establishing a PVC recognizer by matching the correlation coefficients with the template. Zhou et al. [7] proposed a new method for detecting PVC in combination with deep neural networks and rule inference. Llamedo and Martínez [8] studied and validated a simple heartbeat classifier that separates supraventricular and ventricular beats based on RR features and features derived from wavelet transform. Zhang et al. [9] proposed a method of selecting effective feature subsets by one-to-one comparison, using SVM to perform heartbeat classification. Sahoo et al. [10] proposed a method based on multiresolution wavelet transform, which was tested on 48 records of MIT-BIH arrhythmia database to realize classification of four heartbeat types. The ECG waveform is complex. By observing the ECG waveform, it is easy to obtain a large number of ECG features in terms of morphology. However, only a single feature cannot achieve high-precision classification [5, 6, 10], which will reduce the accuracy of classification.

In this paper, a method based on the combination of features and random forest is proposed to distinguish the difference between non-PVC heartbeats and PVC heartbeats. Considering that a single feature may not achieve good results, multiple features will reduce the efficiency of the classifier. Therefore, this paper selected three characteristic parameters (RR intervals, QRS area, and R amplitude) through experimental verification and used them together to detect PVC. The experiment was trained on 22 records in the MIT-BIH arrhythmia database and verified on 22 nonoverlapping records. This method not only reduces the computation time and complexity of the classifier but also achieves a high-precision recognition rate of the datasets.

The outline of this paper is as follows: Section 2 introduces the datasets used in ECG databases and experiments and discusses in detail the methods of detecting PVC, including signal preprocessing, feature extraction, random forest classification learning methods, and processing of unbalanced datasets. Performance evaluation and experimental analysis are explained in Section 3.  Section 4 is the summary of this paper.

## 2. PVC Identification Method Based on Features

The experimental data used in this paper is the publicly available MIT-BIH arrhythmia database [11]. The MIT-BIH arrhythmia database is one of the three most recognized and widely used standard ECG databases in the world and has been widely used in the verification and classification of arrhythmia algorithms. The database consists of a total of 48 records, each containing two signals, each with a length of 650,000 samples and a duration of approximately 30 minutes, sampled at 360 Hz. The 48 records contained 23 records (numbered from 100 to 124 inclusive with some numbers missing) randomly selected from more than 4000 Holter recordings. The remaining 25 records (numbered from 200 to 234 inclusive, including some missing numbers) were the records of uncommon but clinically significant arrhythmias. The MIT-BIH data consists of three parts: the header file [.hea], which is stored in the ASCLL code; the data file [.dat], which is stored in the 212 format; and the comment file [.atr], which also uses binary storage. From the MIT-BIH arrhythmia database record, we know that four of the 48 records (102, 104, 107, and 217) contain paced beats. According to the recommendations recommended by the Association for the Advancement of Medical Instrumentation (AAMI), this paper discards four records and uses the remaining 44 records as experimental data. In this paper, in order to compare with other methods, we divided 44 records into two datasets DS1 and DS2, which are close to 1 : 1. DS1 is used for training, DS2 is used for testing, and each dataset contains 22 records from the ECG database. The specific division is shown in Table 1. Based on the AAMI standard, there are five types of heartbeats: N, S, V, F, and Q. Before entering data into the classifier, mark N, S, F, and Q types as non-PVC types so that the dataset contains only PVC and non-PVC categories, with a focus on PVC category data.

**Figure 1:**
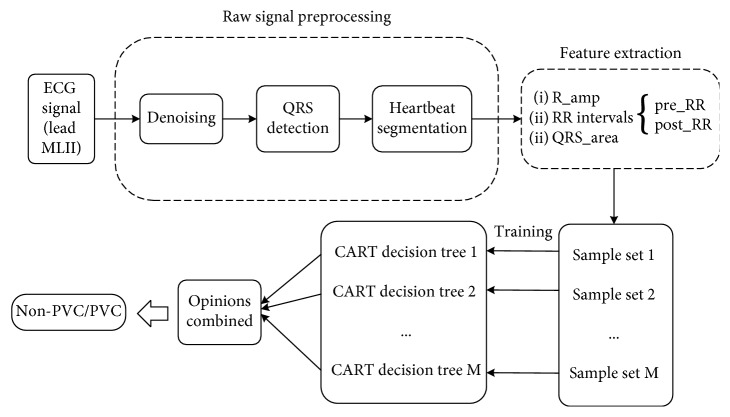
Frame diagram of PVC identification.

Figure 1 shows the various stages of detecting premature ventricular contraction, namely, signal preprocessing, feature extraction, and classification.

### 2.1. Signal Preprocessing

In this paper, the signal preprocessing part is divided into four stages, which consists of the data reading stage, denoising processing stage, QRS identification stage, and heartbeat segmentation stage. The MIT-BIH arrhythmia database consists of 48 two-conductor records, which were only read using MLII lead data during the data read phase. In the process of collecting signals, the original signal was denoised by a wavelet filter, and then the R wave was located by digital analysis of the slope, amplitude, and width. Finally, a single heartbeat was extracted from the complete ECG signal with the R peak as the center. The contents of each stage will be described in detail below.

During the acquisition process, the ECG signal is often interfered by various external noises, which inevitably affects the location of the ECG waveform feature points. In order to correctly identify the waveform and extract more accurate features in later research, the ECG signal must be denoised by the preprocessing process to improve the signal-to-noise ratio.

In recent years, wavelet transform has been widely used in signal denoising research. It is a method that can perform localized signal analysis in both time and frequency domains. It has the characteristics of multiresolution analysis and is suitable for analyzing nonstationary signals and extracting local features of signals. For example, Alyasseri et al. [12] denoised the nonstationary ECG signals by using the *β*-hill climbing metaheuristic algorithm and the wavelet transform method, which achieved good results. Similarly, Wang et al. [13] proposed an adaptive threshold method based on wavelet transform, which also achieves the effect of noise reduction by dynamically adjusting the threshold. Therefore, this paper used Singh and Tiwari [14] to propose an optimal 8-order Daubechies mother wavelet basis function method and performed signal denoising in the wavelet domain. It determines the optimal wavelet filter in three steps: firstly, the base wavelet filter and the low-pass filter are selected from the wavelet filter library; secondly, the correlation coefficient between the ECG signal and the selected wavelet filter is calculated; finally, the number of cross-correlations was maximized to determine the best wavelet filter. This method suppresses the interference of high frequency noise and effectively distinguishes the signal from the noise.

QRS wave recognition plays an important role in ECG signal analysis. Heart rate, RR interval, and morphology can only be calculated after determining the QRS complex, to distinguish between PVC heartbeats and non-PVC heartbeats. There are many algorithms for detecting QRS complexes. Since the “Pan and Tompkins” QRS recognition algorithm based on the differential method [15] is easy to understand and implement, we choose this algorithm to locate the R wave. The algorithm first used a digital band pass filter to reduce false identification due to interference present in the signal and then squared the data samples so that the signal passes through the moving window to obtain waveform characteristics other than the slope of the R wave. Finally, the R wave was identified by digital analysis of slope, amplitude, and width. The algorithm automatically adjusts thresholds and parameters on a regular basis to accommodate changes in QRS morphology and heart rate, providing an accurate method of use for ECG signals with multiple signal characteristics and QRS morphologies [15]. After that, this paper compared the R wave detected by the “Pan and Tompkins” method with the R wave marked in the MIT-BIH arrhythmia database.  Table 2 shows the detailed test results.

After the R wave is detected, a single heartbeat is extracted for ECG signal segmentation. By observing the ECG waveform, the R peak is easier to identify and extract than other features. Therefore, researchers are accustomed to divide a single heartbeat with the R peak as a reference. According to the empirical value, the 100 sampling points before each detected R point are used as the starting point of a single heartbeat, that is, the starting point of the P wave. Similarly, the 150 sampling points after each detected R point are taken as the end point of a single heartbeat, that is, the end point of the T wave. Assuming that the detected R point is R_sample, and the entire heartbeat interval is recorded as [R_sample−100, R_sample+150].

### 2.2. Feature Extraction

In the feature extraction phase, we used 44 recorded data. According to Table 2, the number of wrong heartbeats and the number of missed heartbeats in the 44 records are only 0.54% and 0.37%. Therefore, we excluded the wrong and missing heartbeats and used the remaining heartbeats in the following study. In Section 2.1, a single heartbeat and the start and end points of the heartbeat are extracted, but these few points are not enough to extracte more features. Next the start and end points of the QRS complex will be used to extract the PR interval, QRS interval, and QT interval. In recent years, there are many methods to identify PVC based on PVC disease characteristics, and we used 7 features (R amplitude [16], PR interval, QRS interval, QT interval, QRS area [17], pre_RR interval [18, 19], and post_RR interval [20]) for the study. In order to facilitate the subsequent calculation and understanding, the seven features are, respectively, recorded as R_amp, PR, QRS, QT, QRS_area, pre_RR, and post_RR. In addition, the start and end points of the heartbeat and the start and end points of the QRS complex are also marked as P_start, T_end, QRS_start, and QRS_end.

For the two points QRS_start and QRS_end, the sliding window method is used by us. For example, when looking up the QRS_start point, we narrow down the moving range of the sliding window and define it as [P_start, R_sample]. According to the empirical value, the size *m*  of the sliding window is set to 10. Moving backward from the 4^th^ point after the P_start point (*X*_1_), the interval of the sliding window is [*X*_*i*_ − 4, *X*_*i*_+5], where  1 ≤ *i* ≤ R_sample_ − P_start_ − 8. With each window sliding, the amplitude variance *S*_1_ corresponding to [*X*_*i*_ − 4, *X*_*i*_] and the amplitude variance *S*_2_ corresponding to [*X*_*i*_+1, *X*_*i*_+5] are dynamically calculated according to equation (1). Then, *S*_1_ and *S*_2_ are compared. When *S*_1_ < *S*_2_ and the *S*_1_ value fluctuates around 0, it is judged that the [*X*_*i*_ − 4, *X*_*i*_] segment is a stationary baseline. Then, it is judged whether the five values after the *X*_*i*_ point are rising or falling. If so, the *X*_*i*_ point is regarded as the QRS_start point; otherwise, the window continues to move backward. Similarly, when looking for the QRS_end point, move within the [R_sample, T_end] interval, first find a smooth baseline, and then find the QRS_end point:

(1) $$\begin{array}{c} S^{2}=\frac{\sum_{k=1}^{n}{\left(X_{k}-X\right)}^{2}}{n}, \end{array}$$

where *X*_*k*_ represents the amplitude corresponding to [*X*_*i*_ − 4, *X*_*i*_] or [*X*_*i*_+1, *X*_*i*_+5], *X* represents the average amplitude, and *n* represents the number of points.

**Figure 2:**
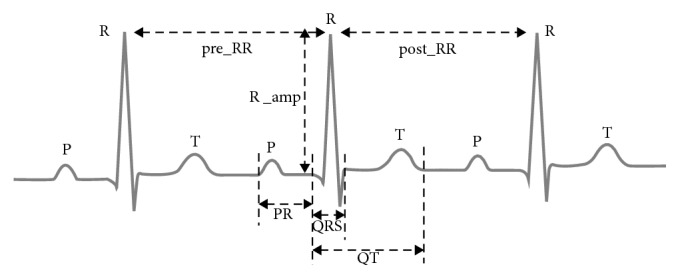
Annotation of heartbeat features.

Using the five points obtained above (P_start, QRS_start, R_sample, QRS_end, and T_end), we can get some of the features used in this study. The following is a detailed description of each feature and the annotation in the signal (Figure 2):

- R amplitude: the amplitude used in the paper is the amplitude after the noise is filtered out by the filter.
- PR interval: the width of the PR interval is defined as the distance from the P_start point to the QRS_start point, so  PR  interval(PR)=(QRS_start_ − P_start_/360). Among them, 360 is the sampling rate used in this paper.
- QRS interval: the width of the QRS interval is defined as the distance from the QRS_start point to the QRS_end point, so QRS interval(QRS)=(QRS_end_ − QRS_start_/360). 360 has the same meaning as expressed in the PR interval.
- QT interval: the width of the QT interval is defined as the distance from the QRS_start point to the T_end point, so  QT interval(QT)=(T_end_ − QRS_start_/360). Similarly, 360 has the same meaning as expressed in the PR interval.
- QRS area: the QRS area is defined as the product of the QRS interval and the R amplitude, so QRS area(QRS_area_)=QRS × R_amp_.
- After extracting a single heartbeat, the above five features can be obtained by sequentially traversing each heartbeat. However, the following two features can be extracted after detecting the R wave.
- pre_RR interval: each heartbeat is based on the R peak; each heartbeat is sequentially traversed to obtain the distance between the current heartbeat and the previous heartbeat and then divided by the sampling rate (360) to obtain the pre_RR interval (pre_RR). It should be noted that starting from the second heartbeat is because the first heartbeat cannot get the previous RR interval.
- post_RR interval: the same method as pre_RR is used to traverse each heartbeat to get the distance between the current heartbeat and the back heartbeat and then divide by the sampling rate to get the post_RR interval (post_RR). It should be noted that the last heartbeat should be discarded because the last heartbeat cannot obtain the subsequent RR interval.

**Figure 3:**
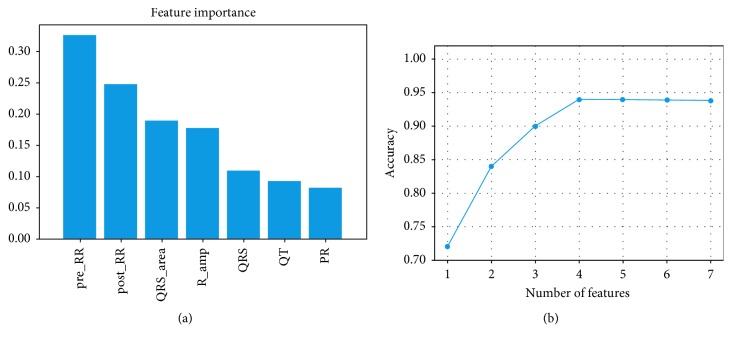
Feature optimization: (a) sorting result of feature importance; (b) effect of different quantitative characteristics on the results.

In the feature extraction stage, seven features are extracted. In order to improve the efficiency of the classifier, the random forest classifier is used to further analyze the impact of different quantitative features on the classification result. The results of the analysis can be seen in Figure 3. We analyse the importance of each feature to the results. One of the seven features was selected to study on the DS1 dataset, and the number of V types that can be correctly identified by using only this feature is obtained. Similarly, another feature is selected for experimentation until all seven features have been selected. We then rank the importance of the classification based on each feature, as shown in Figure 3(a). Among them, pre_RR has the greatest impact on the result and only adopts the pre_RR feature to achieve a recognition rate of 33%. The top rankings are post_RR (24%), QRS_area (18%), and R_amp (17%). At the same time, we have studied different experimental results produced by different numbers of feature subsets on the DS2 dataset. Based on the ranking order obtained in Figure 3(a), we enter different numbers of features in turn to get the results shown in Figure 3(b). When the number of features is only one, the effect is not good, which is within our expectation. As the number of features increases, the classification accuracy continues to increase. When the number of features is increased to 4, the classification effect is obviously good, and the effect is almost the same as when the number of features is 5, 6, and 7. It is further illustrated that these four features play an important role in PVC identification. It is finally concluded that the use of these four features can achieve a good classification effect of the test set. Therefore, the RR interval (pre_RR and post_RR), the QRS region (QRS_area), and the R amplitude (R_amp) are used for the study in Section 3.

### 2.3. Random Forest

**Figure 4:**
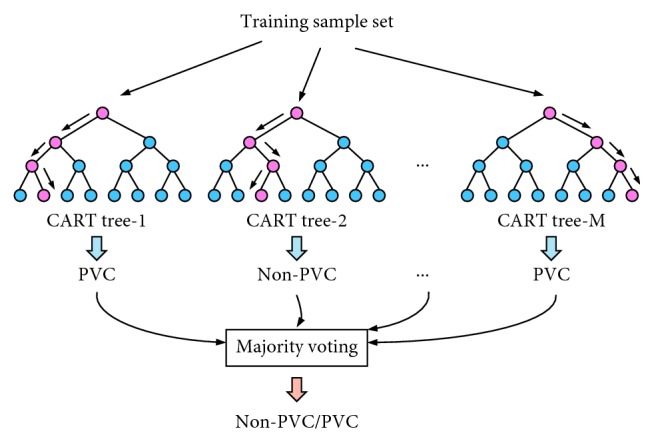
Training model based on random forest (*M* represents the number of trees, and Tree-*M* represents the *M*^th^ tree).

As a highly flexible machine learning algorithm, random forest (RF) [21] has broad application prospects. RF is an important Bagging-based integrated learning method. It contains multiple decision trees and is a combination of several inefficient models into an efficient model. In practical applications, it is widely used in classification and regression problems because of its high accuracy, strong antinoise ability, difficulty in overfitting, ability to process unbalanced datasets [22], and no need to standardize datasets [23]. In this paper, a decision tree based on the CART algorithm was used to construct a random forest classifier. CART (classification and regression tree) is a well-known decision tree learning algorithm, and classification and regression tasks are available. The algorithm that based on the training model of random forest is shown in Figure 4.

Bagging is the most famous representative of the parallel integrated learning method [24]. Its basic process is to sample *T* samples with *m* training samples and then train a base learner based on each sample set. Finally, these base learners are combined. Its algorithm description is shown in Algorithm 1.

*h*_*t*_ represents the  *T*^th^ learner, and *𝒟*_bs _ is the sample distribution generated by self-sampling.

Bagging can be applied to tasks such as two-category, multiclassification, and regression, which is one of its advantages. In addition, it can use the remaining samples in the initial training set as a validation set to perform an “out-of-bag estimate” on generalization performance, recording the training sample used by each base learner. Let *D*_*t*_ denote the training sample set actually used by  *h*_*t*_; let *H*^oob^(*x*) denote the out-of-packet prediction for sample  *x*; that is, only those base learners that do not use *x* training on *X* are considered as

(2) $$\begin{array}{c} H^{\text{oob}}\left(x\right)=\underset{y\in y}{\arg\max}\overset{T}{\underset{t=1}{\sum }}\left(h_{t}\left(x\right)=y\right)\cdot \prod^{}\left(x\notin D_{t}\right). \end{array}$$

Then, the out-of-package estimate of bagging generalization error is

(3) $$\begin{array}{c} \varepsilon^{\text{oob}}=\frac{1}{\left|D\right|}\underset{\left(x,y\right)\in D}{\sum }\prod^{}\left(H^{\text{oob}}\left(x\right)\neq y\right). \end{array}$$

The CART decision tree [25] used in this paper uses the “Gini index” to select the partitioning attribute. Assuming that the proportion of the *k*^th^ sample in the current sample set *D* is  *p*_*k*_(*k*=1,2,…, *K*), the purity of the dataset *D* can be measured by the Gini value:

(4) $$\begin{array}{c} \text{Gini}\left(D\right)=\overset{K}{\underset{k=1}{\sum }}\underset{k^{\prime }\neq k}{\sum }p_{k}p_{k^{\prime }}=1-\overset{K}{\underset{k=1}{\sum }}p_{k}^{2}. \end{array}$$

Gini(*D*)  reflects the probability of inconsistent classification of two samples from dataset *D*. Taking the two categories, we studied as an example, if the sample categories to be extracted are of the same category, the Gini(*D*)=1 − ∑_*k*=1_^*K*^*p*_*k*_^2^=0. If the sample categories extracted are of two types and the numbers are the same, the Gini(*D*)=1 − ∑_*k*=1_^*K*^*p*_*k*_^2^=1/2. Therefore, the smaller the Gini(*D*), the higher the purity of the dataset  *D*.

Suppose the discrete attribute *a*  has *V*  possible values  {*a*^1^, *a*^2^,…, *a*^*V*^}. If *a* is used to divide the sample set  *D*, *V* branch nodes are generated. The  *V*^th^ branch node contains all samples in  *D* that have a value of *a*^*v*^ on attribute *a*, which is denoted as  *D*^*v*^. We calculated the Gini value of *D*^*v*^ according to equation (4) and then gave the branch node a weight |*D*^*v*^|/|*D*|, considering that the number of samples included in different branch nodes is different. That is, the more the number of samples, the greater the influence of the branch nodes, so the Gini index of the attribute *a* is defined as

(5) $$\begin{array}{c} \text{Gini\_index}\left(D,a\right)=\overset{V}{\underset{v=1}{\sum }}\frac{\left|D^{v}\right|}{\left|D\right|}\text{Gini}\left(D^{v}\right). \end{array}$$

Then, in the candidate attribute set *A*, we selected the attribute that makes the postpartition Gini index the smallest as the optimal partition attribute, i.e.,

(6) $$\begin{array}{c} a_{*}=\underset{a\in A}{\arg\;\min}\text{Gini\_index}\left(D,a\right). \end{array}$$

**Figure 5:**
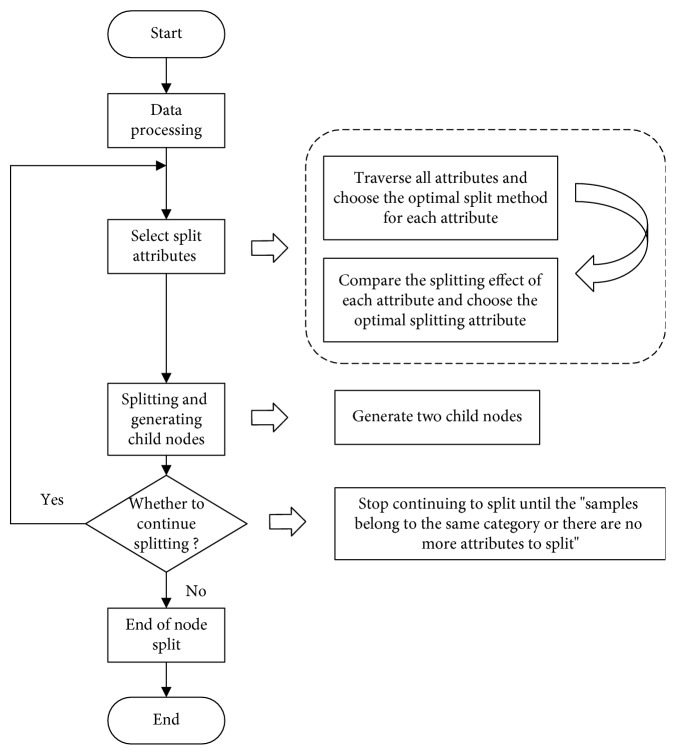
Flow chart of attribute division.

The detailed flow chart of attribute division is shown in Figure 5.

### 2.4. Synthetic Minority Oversampling Technique Algorithm

A serious imbalance in the dataset is studied in this paper. It can be seen in Table 1 that the ratio of the non-*V* type to *V* type is about 14 : 1 (44 recordings), which will undoubtedly affect the results of the study. In order to avoid this, the SMOTE (Synthetic Minority Oversampling Technique) algorithm is chose to manually synthesize new samples from minority class samples. The specific process of the algorithm is divided into three steps:

- Suppose *x*  is each sample in a minority class and *S*  is a minority class sample set. Calculate the distance *d* from *x* to all samples in *S* using the Euclidean distance (equation (7)) and get its *k*  nearest neighbors:

(7) $$\begin{array}{c} d=\sqrt{\overset{n}{\underset{i=1}{\sum }}{\left(a_{i}-b_{i}\right)}^{2}}, \end{array}$$

  where *a*_*i*_ represents the *i*^th^ dimension of *x*  and *b*_*i*_ represents the *i*^th^ dimension of a certain sample in  *S*.
(b) Determine the sampling magnification *N* according to the sample imbalance ratio and randomly select several samples from its *k* nearest neighbors for *x*.
(c) Assuming that the randomly selected neighbor is  *x*′, each selected  *x*′ and *x*, respectively, construct a new sample *x*_new _ according to the following equation:

(8) $$\begin{array}{c} x_{\text{new}}=x+r\text{ and }\left(0,1\right)\times \left(x^{\prime }-x\right). \end{array}$$

## 3. Experiment

### 3.1. Evaluation Indicators

In this paper, accuracy (Acc), positive predictive value (PPV), sensitivity (Se), specificity (Sp), and *γ* (Youden's index [26]) are used as evaluation indicators for the algorithm. Accuracy is the most commonly used metric, which refers to the ratio of the number of samples correctly classified by the classifier to the total number of samples for a given test dataset. Generally speaking, the higher the correct rate, the better the classification effect. The positive predictive value indicates the proportion of the number of cases that are truly positive among the total number of positive cases tested. Sensitivity represents the proportion of positive cases that are correctly identified in all practical positive cases. Specificity represents the proportion of negative cases that are correctly identified in all actual negative cases. In addition to the above evaluation indicators, this paper also used a combined result of sensitivity and specificity, namely, *γ*. The above indicator equations and the confusion matrix of the classification (Table 3) are as follows:

(9) $$\begin{array}{c} \text{Acc}=\frac{\text{TP}+\text{TN}}{\text{TP}+\text{TN}+\text{FP}+\text{FN}},\\ \text{PPV}=\frac{\text{TP}}{\text{TP}+\text{FP}},\\ \text{Se}=\frac{\text{TP}}{\text{TP}+\text{FN}},\\ \text{Sp}=\frac{\text{TN}}{\text{TN}+\text{FP}},\\ \gamma =\text{Se}+\text{Sp}-1. \end{array}$$

### 3.2. Experiment and Result Analysis

Before the experiment, we synthesized the new samples for the DS1 dataset using the algorithm in Section 2.4 and placed the new samples in the original datasets and then used the expanded dataset for research. After adding new samples, the ratio of non-*V* and *V* in the DS1 dataset is close to 1 : 1.

#### 3.2.1. Analysis of Experimental Results of Different Parameters

Since random forest is random, when we set different values for parameters, it produces different classification results. For example, *n*_estimators represents the maximum number of decision tree. In general, the value is too small, it is easy to under fitting, its value is too large, and it is easy to overfitting, so choosing a suitable value is crucial for research. Based on the different *n*_estimators values, we studied and analyzed the DS1 dataset, as shown in Table 4.

Obviously, as can be seen from Table 4, the DS1 dataset achieves the best RF performance at *n*_estimators = 120. Its indicators are 98.29%, 97.58%, and 96.58%. This indicates that different parameters have an effect on the experimental results. After that, we adjusted the other parameters of the DS1 dataset and got the optimal parameters on the classifier (DS1: *n*_estimators = 120, min_samples_split = 100, and min_samples_leaf = 30).

#### 3.2.2. Analysis of Experimental Results of Various Classifiers

In this paper, five evaluation indicators of Acc, PPV, Se, Sp, and *γ* are used to compare the performance differences among *K*-nearest neighbor (KNN), logistic regression (LR), naive Bayes (NB), multilayer perceptron (MLP), decision tree (DT), and random forest (RF) on the unbalanced binary dataset (DS2).  Table 5 presents a performance comparison of the various classifiers.

According to the results in Table 5, the results obtained using the NB algorithm are relatively the worst. The NB's indicators are the lowest, indicating that the method may not be applicable to the dataset. The best result is RF. In addition to the lowest PPV (95.46%), the four indicators of RF (Acc, Se, Sp, and *γ*) show the best results. RF is composed of multiple decision trees, and its final result is determined by all decision tree voting. This is the biggest advantage of RF compared with other algorithms. Therefore, its classification effect is better than the other five methods, which is why we choose random forest instead of other classifiers.

#### 3.2.3. Analysis of Experimental Results of Unbalanced Datasets

Due to the large differences in the types of datasets, we conducted experiments on this issue. On the initial unbalanced training set DS1 (13 : 1), the new data were synthesized using the SMOTE algorithm, which made the non-V type and V type in the DS1 dataset reach 8 : 1, 4 : 1, and 1 : 1, respectively. We trained with different proportions of DS1 datasets and tested them on the unbalanced training set DS2. From Table 6, we can see that, as the ratio between non-*V* and *V* shrinks, the results are getting better and better. The value of each indicator is constantly changing, with the value of *γ* changing the most (37.13%), rising from 58.32% to 95.45%, and the other indicators are rising by 17%–20%. This undoubtedly shows that an unbalanced dataset can have a serious impact on the experiment.

#### 3.2.4. Comparative Analysis with Other Methods

Table 7 shows the evaluation of PVC performance between the methods that are used in this paper and the research methods of other scholars. This paper takes the same test set [5–9, 27, 28], as the study object, and compares the results of other publications in different records. As can be seen from the experimental results of the seven records, the proposed classifier of this paper achieved better positive predictive value (99.44%) and specificity (99.45%). Its accuracy (99.32%) is only 0.03% lower than 99.35% of [5]. Similarly, 6 records in the ECG are used to compare with others. The results showed that the sensitivity (99.09%), specificity (99.34%), and *γ* (98.43%) of the proposed method were not the best, but the accuracy and positive predictive value were better than any results. Finally, we applied the parameters obtained in Section 3.2.1 to DS1 for training and then tested the classifier on DS2. The results of the classifier for these records are accuracy (96.38%), positive predictive value (95.46%), sensitivity (97.88%), specificity (97.56%), and *γ* (95.45%). It is clear that accuracy (96.38%) and specificity (97.56%) are lower than other results. However, the other three indicators showed the best results, of which *γ* (95.45%) was second only to the result of [7] (97.13%). The final experimental results show that the method of this paper has a good effect on identifying PVC.

## 4. Conclusions

Automatic analysis technology for identifying PVC has been established in the field of ECG research for several decades. In order to improve the identification rate of PVC, many scholars have been exploring this aspect. This paper proposes a method based on a combination of multiple features and a random forest algorithm to distinguish PVC. PVC can be identified by the characteristics of RR intervals (pre_RR and post_RR), R amplitude, and QRS area. These features were tested for 22 records (DS2) in the MIT-BIH arrhythmia database and achieved good results. The accuracy, positive predictive value, sensitivity, specificity, and *γ* of the method reach 96.38%, 95.46%, 97.88%, 97.56%, and 95.45%, respectively. The results demonstrate that the method has a high recognition rate in ECG data, which makes a great significance in clinical application. However, this method is currently only validated in two types of studies. For the problem of multicategory research, it is also a problem that needs more time to explore in the future.

## Figures and Tables

**Algorithm 1:**
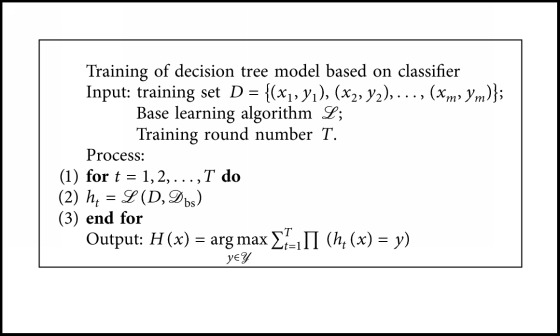
Algorithm description of decision tree.

**Table 1:** The method of detailed division of datasets.

Dataset	Recordings	Division method	Non-V	V	Total
DS1	101, 106, 108, 109, 112, 114, 115, 116, 118, 119, 122, 124, 201, 203, 205, 207, 208, 209, 215, 220, 223, 230	Training	47573	3648	51221
DS2	100, 103, 105, 111, 113, 117, 121, 123, 200, 202, 210, 212, 213, 214, 219, 221, 222, 228, 231, 232, 233, 234	Test	46539	3157	49696
DS1 + DS2	44 recordings	—	94112	6805	100917

*Note.* Non-*V* represents non-PVC type, and V represents PVC type.

**Table 2:** Comparison of R waves marked in MIT-BIH and R waves detected by “Pan and Tompkins.”

Records	All	Correct	Wrong	Missed	Se	P + (PPV)
100	2273	2272	0	1	0.9996	1
101	1865	1865	5	0	1	0.9973
103	2084	2084	0	0	1	1
105	2572	2570	44	2	0.9992	0.9832
106	2027	2018	3	9	0.9956	0.9985
108	1763	1746	61	17	0.9904	0.9662
109	2532	2532	7	0	1	0.9972
111	2124	2123	4	1	0.9995	0.9981
112	2539	2539	3	0	1	0.9988
113	1795	1794	0	1	0.9994	1
114	1879	1878	4	1	0.9995	0.9979
115	1953	1953	0	0	1	1
116	2412	2391	3	21	0.9913	0.9987
117	1535	1535	1	0	1	0.9993
118	2278	2278	12	0	1	0.9948
119	1987	1987	0	0	1	1
121	1863	1863	3	0	1	0.9984
122	2476	2476	0	0	1	1
123	1518	1515	0	3	0.9980	1
124	1619	1618	0	1	0.9994	1
200	2601	2600	63	1	0.9996	0.9763
201	1963	1948	0	15	0.9924	1
202	2136	2128	0	8	0.9962	1
203	2980	2967	89	13	0.9956	0.9709
205	2656	2653	0	3	0.9989	1
207	2332	2135	22	197	0.9155	0.9898
208	2955	2941	10	14	0.9953	0.9966
209	3005	3005	4	0	1	0.9987
210	2650	2604	15	46	0.9826	0.9943
212	2748	2748	0	0	1	1
213	3251	3250	0	1	0.9997	1
214	2262	2259	6	3	0.9987	0.9974
215	3363	3363	1	0	1	0.9997
219	2154	2154	0	0	1	1
220	2048	2048	0	0	1	1
221	2427	2422	0	5	0.9979	1
222	2483	2482	5	1	0.9996	0.9980
223	2605	2604	2	1	0.9996	0.9992
228	2053	2051	166	2	0.9990	0.9251
230	2256	2256	1	0	1	0.9996
231	1571	1570	0	1	0.9994	1
232	1780	1780	14	0	1	0.9922
233	3079	3073	1	6	0.9981	0.9997
234	2753	2751	0	2	0.9993	1
Total	**101205**	**100829**	**549**	**376**	**0.9963**	**0.9941**

*Note.* The first column is the name of the record, the second column is the number of R waves marked in MIT-BIH, the third column is the number of correctly detected R waves, the fourth column is the number of falsely detected R waves, the fifth column is the number of missed R waves, the sixth column is the evaluation indicator—sensitivity, and the seventh column is the evaluation indicator—positive prediction rate. According to the AAMIEC38 standard, the difference between the detected QRS complex and the manual mark is within 150 ms, which means that the location detection is successful.

**Table 3:** Classification of confusion matrix.

		Forecast category	
		N	V
Actual category	N	TN	FP
	V	FN	TP

*Note. N* represents non-PVC type, and *V* represents PVC type.

**Table 4:** Effect of different *n*_estimators values on classification results.

Parameter	DS1		
*n*_estimators	Acc (%)	PPV (%)	*γ* (%)
10	97.97	97.81	95.95
60	98.18	97.57	96.37
100	98.22	97.71	96.44
**120**	**98.29**	**97.58**	**96.58**
150	98.17	97.50	96.33

**Table 5:** Comparison of experimental results of 6 classifiers.

Classifier	Acc (%)	PPV (%)	Se (%)	Sp (%)	*γ* (%)
KNN	95.04	96.79	97.27	97.26	94.55
LR	95.82	96.10	97.46	97.45	95.10
NB	93.59	95.70	96.05	96.06	92.11
MLP	96.22	97.26	97.77	97.02	94.99
DT	94.62	96.38	96.18	96.36	92.54
**RF**	**96.38**	**95.46**	**97.88**	**97.56**	**95.45**

**Table 6:** Impact of unbalanced datasets on experiments.

Proportion	Acc (%)	PPV (%)	Se (%)	Sp (%)	*γ* (%)
13 : 1	77.34	75.01	80.10	78.21	58.32
8 : 1	84.20	84.38	85.00	86.92	71.93
4 : 1	89.47	90.63	92.50	92.08	84.59
1 : 1	96.38	95.46	97.88	97.56	95.45

**Table 7:** Comparison with other literatures.

Methods	Recordings	Measures				
		Acc (%)	PPV (%)	Se (%)	Sp (%)	*γ* (%)
Shyu et al. [27]	111, 115, 116, 119, 221, 230, 231	97.04	—	99.02	—	—
Li et al. [6]		98.0	66.0	99.7	—	—
Zarei et al. [5]		99.35	90.94	100	99.33	99.33
Proposed method		**99.32**	**99.44**	**99.19**	**99.45**	**98.64**

Lim [28]	115, 116, 119, 221, 230, 231	99.8	—	99.2	—	—
Li et al. [6]		99.7	79.0	99.6	—	—
Zarei et al. [5]		99.74	96.65	100	99.72	99.72
Proposed method		**99.81**	**99.34**	**99.09**	**99.34**	**98.43**

Llamedo and Martínez [8]	All recordings of DS2	98.16	87.97	82.94	99.21	82.15
Zhang et al. [9]		98.63	92.75	85.48	99.54	85.02
Zarei et al. [5]		98.77	86.48	96.12	98.96	95.08
Zhou et al. [7]		99.41	93.55	97.59	99.54	97.13
Proposed method		**96.38**	**95.46**	**97.88**	**97.56**	**95.45**

## Data Availability

All datasets used to support the findings of this study are included within the article. All datasets used to support the findings of this study were supplied by the publicly available MIT-BIH database from the Massachusetts Institute of Technology. The URL to access the data is https://www.physionet.org/cgi-bin/atm/ATM. The coding used to support the findings of this study have not been made available because the source code in this article is part of a national project and is a trade secret, and the source code is not available.
